# Carbon nanotubes for rapid capturing of SARS-COV-2 virus: revealing a mechanistic aspect of binding based on computational studies

**DOI:** 10.1039/d0ra08888a

**Published:** 2021-02-02

**Authors:** Shivkumar Patel, Amit Kumar Srivastav, Sanjeev K. Gupta, Umesh Kumar, S. K. Mahapatra, P. N. Gajjar, I. Banerjee

**Affiliations:** School of Nano Sciences, Central University of Gujarat Gandhinagar 382030 India indrani.banerjee@cug.ac.in; Computational Materials and Nanoscience Group, Department of Physics, St. Xavier's College Ahmedabad 380009 India sanjeev.gupta@sxca.edu.in; Department of Physics, Central University of Punjab Bathinda 151001 India; Department of Physics, University School of Sciences, Gujarat University Ahmedabad 380009 India

## Abstract

We investigate the binding interactions of synthesized multi-walled carbon nanotubes (MWCNTs) with SARS-CoV-2 virus. Two essential components of the SARS-CoV-2 structure *i.e.*6LU7 (main protease of SARS-CoV-2) and 6LZG (spike receptor-binding domain complexed with its receptor ACE2) were used for computational studies. MWCNTs of different morphologies (zigzag, armchair and chiral) were synthesized through a thermal chemical vapour deposition process as a function of pyrolysis temperature. A direct correlation between radius to volume ratio of the synthesized MWCNTs and the binding energies for all three (zigzag, armchair and chiral) conformations were observed in our computational studies. Our result suggests that MWCNTs interact with the active sites of the main protease along with the host angiotensin-converting enzyme2 (ACE2) receptors. Furthermore, it is also observed that MWCNTs have significant binding affinities towards SARS-CoV-2. However, the highest free binding energy of −87.09 kcal mol^−1^ with 6LZG were shown by the armchair MWCNTs with SARS-CoV-2 through the simulated molecular dynamic trajectories, which could alter the SARS-CoV-2 structure with higher accuracy. The radial distribution function also confirms the density variation as a function of distance from a reference particle of MWCNTs for the study of interparticle interactions of the MWCNT and SARS-CoV-2. Due to these interesting attributes, such MWCNTs could find potential application in personal protective equipment (PPE) and diagnostic kits.

## Abstract 2

Investigation of the binding interactions of synthesized multi-walled carbon nanotubes (MWCNTs) with SARS-CoV-2 virus.

## Graphical abstract

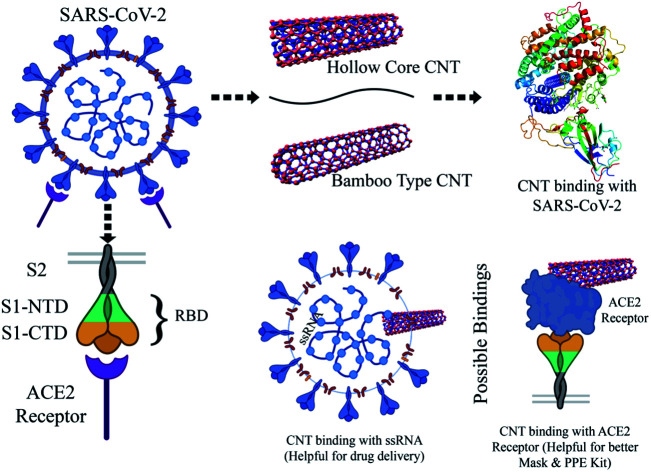

## Introduction

SARS-CoV-2 virus has evolved so rapidly and unpredictably that it has challenged the present high-tech medical technology for effective disease diagnosis and therapy. The scientific society is vigorously working on SARS-CoV-2 virus vaccines. But to date only a few vaccines have been approved around the world; many more are still in the development phase. Yet, this pandemic is still killing a huge proportion of society globally. To help control the outbreak, preventive measures in terms of rapid capturing of the virus in the near vicinity and its entrapment before coming into direct contact with humans could be helpful in the present scenario. This virus is known to spread primarily *via* fomite and droplet transmission that can spread to longer distances when aerosolized.^1^ These aerosols possess high affinity towards surface atoms. Moreover, the virus is also spreading asymptomatically by human to human transmission and seems to undergo repeated mutation resulting in considerable morbidity and mortality globally. CNTs have been potentially used as drug delivery vehicles,^2^ bio imaging probes,^3^ bio sensors,^4^ X-ray sources,^5^ neuron protection^6^ and substrates for immunological isolation device for mammalian,^7^ cells bacteria,^8^ viruses.^9,10^ Many works show a great potential in CNTs as a sensors or biosafety material for the detection of SARS-CoV-2 virus.^11,12^ However, the potential of CNT as entrapping the most dangerous virus of the present day *i.e.* SARS-CoV-2 has not been reported yet. Surface bound thermal chemical vapour deposition (CVD) is one of the most conventional method for synthesis of carbon nano structures. Synthesis of CNFs (Carbon Nano Fibers) and CNTs *via* CVD is a catalyst driven pyrolytic chemical synthesis process where the kinetics of carbon supply during pyrolysis process plays crucial role.^13^ MWCNTs of different morphologies (armchair, zigzag, chiral) were synthesized through thermal CVD process as a function of pyrolysis temperature. A carbon nanotube has three different chirality like nanotube is chiral if it has type (*n*,*m*), with *m* > 0 and *m* ≠ *n*; otherwise it is “zigzag” tubes if its chirality is (*n*,0) and for (*n*,*n*) chirality, it will be “armchair” tubes. The present work aims to understand the interaction of MWCNT with that of the SARS-CoV-2 virus in its vicinity. The impact of chirality and other morphology of the MWCNTs on SARS-CoV-2 structure were investigated using molecular docking method and atomistic molecular dynamic (MD) simulation. Through molecular docking studies, we can explore the molecular interaction behavior between different amino acid of targeted virus and potential ligand.^14,15^ For comparison, the molecular docking investigations were also performed using single walled carbon nanotubes. Interestingly, in our molecular docking studies, MWCNTs showed higher affinity towards the SARS-CoV-2 virus as compared to single walled CNTs of same morphology and diameter. Along with molecular docking studies, dynamic behavior, thermodynamic properties, radial distribution function of the hollow and bamboo shaped MWCNTs are analyzed and investigated with SARS-CoV-2 spike RBD complexed with ACE2 receptor through atomistic MD simulation. Interestingly, the highest binding affinity of the MWCNTs was observed towards the 6LZG, 6LU7 motifs of the virus which mainly helps it in cell internalization.^16^ Though, our results seems very promising that MWCNTs has a great potential for rapid capturing of the virus and prevention of cell internalization but it needs to be validated by proper wet lab experimental set up.

The work reveals the binding affinity of synthesized MWCNTs of different morphology with SARS-CoV-2 virus. Our study gives a potential candidate for the rapid capturing and entrapment of the virus within the 3D structure of the MWCNTs, which prevents direct contact of virus with the human body. Such studies can help in designing MWCNT based devices (personal protective equipment and diagnostics) for preventing SARS-COV-2 outbreak in many folds.

## Materials and methods

### Synthesis and characterization of carbon nanotubes

The CNTs were grown using conventional thermal chemical vapour deposition method on 5% by weight of iron catalyst mixed with SiO_2_ at different temperatures of 750 °C, 800 °C and 850 °C. The catalyst was prepared by wet-impregnation method.^17^ The eutectoid temperature of Fe–C being 740 °C, the growth temperature chosen in the present work is of 750 °C and above.^18^ The synthesis is done in a quartz boat containing 0.25 gm of catalyst kept inside the quartz reactor. N_2_ gas was passed through the quartz reactor and simultaneously the furnace temperature was increased up to 580 °C at the rate of around 150 °C per hour. The reduction temperature of 580 °C was maintained and N_2_ was subsequently replaced by H_2_ with the flow rate of 30 ml min^−1^ for 30 minutes. The CNT growth was performed at three different temperatures of 750 °C, 800 °C and 850 °C. When the adequate temperature is reached, the condition was maintained for 30 minutes in the presence of N_2_ (150 ml min^−1^) and H_2_ (30 ml min^−1^) mixture. Simultaneously, carbon source xylene (purchased from Srl Pvt. Ltd.) was injected at the rate of 0.5 ml min^−1^ for 30 min of duration. Due to high temperature in the reactor, xylene got vaporized and decomposed on the catalyst surface, and from there the growth of nanotubes initiates. After 30 minutes, H_2_ flow was stopped and the temperature was allowed to reduce gradually to room temperature and samples were collected and presented for the characterization. The carbon precursor adsorbed by the hot catalyst surface, get dissolved onto the catalyst surface and precipitation of carbon species in the form of CNTs occurs on (base growth) or below (tip growth) the metal particle through vapor–liquid–solid (VLS) mechanism.^19–21^ In this model, the catalysts are in liquid state which preferentially adsorb the carbon species from its surrounding vapours. Solid whiskers are then formed from the supersaturated eutectic liquid.^21^

The crystallographic analysis of the synthesized samples was done by powder XRD of model no. GNR APD 2000 PRO. The XRD spectra was recorded using the Cu K_α_ radiation of wavelength 1.54 Å at a full scan of 10° to 80° of 2*θ* value at the normal scan rate of 1.5° min^−1^. The morphological studies of sample were done by using field emission scanning electron microscopy (FE-SEM) (Model – Merlin Compact by Carl Zeiss) and high resolution transmission electron microscopy (HRTEM) (JOEL JEM 2100). Raman spectroscopy (Model – Renishaw inVia Raman Microscope) was used for studying the defects level of synthesized carbon materials.

### Molecular docking studies

**Fig. 1:**
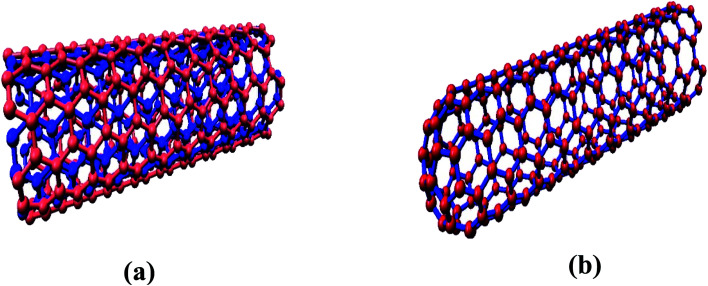
(a) Hollow-core MWCNT structure (b) bamboo type MWCNT.

**Fig. 2:**
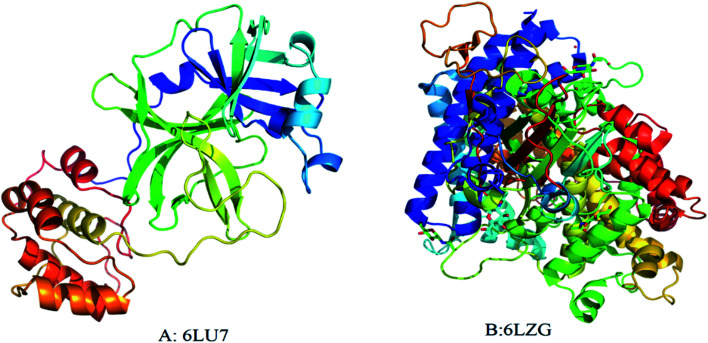
3D structure of original SARS-CoV-2 (2019-nCoV) (A) PDB (6LU7); (B) PDB(6LZG). Each color represents specific amino acid residues. Like bright red indicates ASP, GLU; yellow indicates CYS, MET; blue indicates LYS, ARG; orange represents SER, THR; mid blue indicates PHE, TYR; cyan indicates ASN, GLN; light grey represents GLY; green indicates LEU, VAL, ILE; dark grey indicates ALA; pink indicates TRP; pale blue represents HIS; flesh color indicates PRO amino acids.

The interaction of MWCNTs with SARS-CoV-2 structure for targeting the active sites of the main protease along with the host receptor angiotensin-converting enzyme2 (ACE2) receptor was investigated. The impact of chirality of the MWCNTs on SARS-COV-2 structure was analyzed using molecular docking method. As per experimental analysis of HRTEM, two types of CNT structure were observed in our synthesized CNT *i.e.* hollow core and bamboo type MWCNT. Therefore, we performed molecular docking studies by analysing the effect of both types of MWCNT structure on SARS-CoV-2. Comparing the effect of both type of MWCNT structure on SARS-CoV-2 will give fruitful insight of the activity of main protease and ACE2 receptor for finding the drug binding sites and residues on SARS-CoV-2. The 3D structures of both hollow core MWCNT (HMWCNT) and bamboo type MWCNT (BMWCNT) (Fig. 1) were prepared by Nanotube Modeler software^22^ and their structural parameters are shown in Table 1. Here, atom coordinates are generated using purely geometrical criteria without any saturation method for the edges of the nanotube and not involve any energy calculation. Two different essential components of the SARS-CoV-2 structure *i.e.*6LU7 (COVID-19 main protease) and 6LZG (spike receptor-binding domain complexed with its receptor ACE2) were used from the PDB database for docking studies^23,24^ (Fig. 2). AutoDock4 and Auto Dock Vina package with AMBER force field was used to investigate the molecular interaction between different MWCNT and SARS-CoV-2 virus.^25^ The Lamarckian genetic algorithm was used to analyse the probability of docking studies.^26^ Autodock, Pymol and VMD softwares used for the analysis of the results.

**Table 1:** Structural parameters of the modeled MWCNTs

Multi walled carbon nanotube (length 1 nm)									
	Armchair			Chiral			Zigzag		
*n*	4	5	6	4	5	6	4	5	6
*m*	4	5	6	2	3	3	0	0	0
Atoms	148	183	217	114	146	164	90	110	130
Bonds	204	153	300	155	198	222	117	143	169
Inner radius (Å)	2.714	3.392	4.071	2.073	2.742	3.109	1.567	1.959	2.35
Outer radius (Å)	3.0595	3.737	4.4145	2.4465	3.109	3.4815	1.9585	2.3505	2.742
Outer diameter (Å)	6.119	7.474	8.829	4.893	6.218	6.963	3.917	4.701	5.484
Inner diameter (Å)	5.428	6.785	8.142	4.146	5.484	6.218	3.134	3.917	4.701
Volume (Å)	62.7	77.3	91.6	53	67.5	77.1	43.4	53	62.7
Surface to volume ratio	6	6	6	5.6	5.6	5.6	5.3	5.3	5.3
Radius (I) to volume ratio	0.043285	0.043880	0.044443	0.039113	0.040622	0.040322	0.036105	0.036962	0.037480
Radius (O) to volume ratio	0.048795	0.048344	0.048193	0.046160	0.046059	0.045155	0.045126	0.044349	0.043732

### Molecular dynamic simulation

We performed the atomistic (AA) molecular simulation to understand the interaction between the molecules of a SARS-CoV-2 spike RBD complexed with ACE2 receptor and MWCNT. The initial coordinate of crystallized structure of SARS-CoV-2 spike RBD complexed with ACE2 receptor (resolution: 2.50 Å) was obtained from the RCSB database (PDB: 6LZG).^27^ The SARS-CoV-2 spike RBD complexed ACE2 receptor structure contains two side chains A and B having 596 and 195 sequence of amino acid residues respectively in the crystallographic asymmetric structure database.^27^ We performed MD simulation of both hollow and bamboo shaped MWCNTs based on their chirality. Hollow and bamboo shaped MWCNTs of armchair (6,6), zigzag (6,0), and chiral (6,3) were taken to perform the MD simulation study with the SARS-CoV-2 structure. We kept all MWCNTs with the length of 5.0 nm during simulation study. All MD simulation were carried out by the GROMACS 2020.3 software^28^ at 300 K and 1 atm pressure in NVT and NPT ensembles^29^ using the AMBER FF99SB force field.^30^ All the system of MWCNT and SARS-CoV-2 were solvated in a rectangular box with water. The size of the box was set at 110 Å × 110 Å × 240 Å so that structure could get enough space to relax during the simulation process.^27^ TIP3P water model was used in all the simulations.^31^ Each system was neutralized with specific amount of NaCl counter ions. PME mesh algorithm was used for periodic boundary conditions.^32^ To calculate the long range electrostatic interactions PME mesh algorithm was used at 1 fs time step. The integration step for all atomistic MD simulations was set to 2 fs. The trajectories of the each simulated complex system were saved at 1 ps time interval for capturing the precise and accurate dynamic behavior of the simulated complex structure. All bond lengths involving hydrogen atoms in the complexed structure were constrained by using the SHAKE algorithm.^33^ We performed 50 ns long all atom MD simulation to analyze the structural, thermodynamic behavior of SARS-CoV-2 spike RBD complexed ACE2 receptor with different chirality based MWCNT. The motion trajectories of the simulated complex system were relevant for the calculation of binding energies and radial distribution function.^34,35^ The stability and equilibrium of each simulated complexed structure was ensured by the RMSD, secondary structure and Ramachandran plot.

### Free energy calculations from molecular dynamics simulation studies

The MM/PB(GB)SA method is applied to analyze and calculate the binding free energy of the simulated complexed structure.^36^ The protocols of MM/PB(GB)SA methodology for calculating the binding free energy includes in the MD simulations.^37,38^

In thermodynamic study, the g_mmpbsa function was applied for the calculation of binding free energy of SARS-CoV-2 main protease and ACE2 receptor with MWCNT complexed systems.^38^ Approximately five thousand snapshots were taken at 10 ns time interval to calculate and analyze the MM/PBSA free energy throughout the MD simulation trajectory. For each snapshot of trajectories, eqn (1) was applied to calculate the binding free energy of the receptor–ligand complex system. The binding free energy calculated according to the following equations:

1 Δ*G*_binding_ = *G*_complex_ − (*G*_receptor_ + *G*_nanotube_)

2 Δ*G* = Δ*G*_gas_ + Δ*G*_solv_ − *T*Δ*S*

3 Δ*G*_gas_ = Δ*E*_electrostatic_ + Δ*E*_vdw_

4 Δ*G*_solv_ = Δ*G*_GB_ + Δ*G*_SA_

5 Δ*G*_SA_ = *γ* × ΔSASA

where, *G*_complex_ is the free energy of receptor–ligand complex system. *G*_protein_ is the free energy of receptor system, and *G*_ligand_ is the free energies of the ligand. Δ*G*_gas_ is the gas-phase free energy of molecular mechanics. Δ*E*_electrostatic_ represents the electrostatics energy and in same way Δ*E*_vdw_ represented the van der Waals energy forces. Δ*G*_solv_ represents the solvation free energy of the simulated system, which includes the polar (Δ*G*_GB_) and nonpolar (Δ*G*_SA_) solvation energy calculated through generalized-Boltzmann (GB) equation with dielectric constants of solute and solvent *ε*_w_ = 80 and *ε*_p_ = 1.0 respectively.^39^ Δ*G*_SA_ represents the free energy of solvent accessible surface area. The solvent-accessible surface area (SASA) calculated by linear combinations of pairwise overlaps. Here, *γ* denotes the surface tension, which was set to 0.0072 kcal mol^−1^ Å^−2^.^39,40^*T*Δ*S* represents the entropy value of the simulated system. 5000 snapshots were taken from the 50 ns trajectories of the SARS-CoV-2 spike RBD complexed ACE2 receptor and MWCNT complex systems.

### Radial distribution function

We calculated the radial distribution functions (RDFs) between the carbon (C–C) atoms of different chirality (6,0), (6,3), (6,6) of HMWCNT and BMWCNT with C atom of SARS-CoV-2. The radius of each chirality is shown in Table 1 and the length of MWCNT was kept at 5 nm. The radial distribution function describes about the interparticle interactions of the MWCNT and SARS-CoV-2 with the solvent in terms of thermodynamic properties. We computed two-dimensional RDF *g*(*r*) of simulated complex structure. The radial distribution function *g*(*r*) was computed through the number of particles of given MWCNT on average *n*(*r*, *r* + *d*_r_) in a distance interval (*r*, *r* + *d*_r_) from the MWCNT axis in space as;6
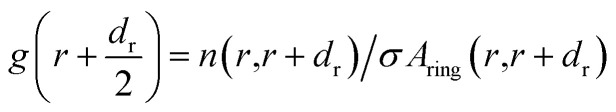

7 *A*_ring_(*r*,*r* + *d*_r_) = π(*r* + *d*_r_)^2^ − π*r*^2^ ≈ 2π*rd*_r_

where *σ* is the area density of the atomic particle and *A*_ring_ is the area of a ring of MWCNT around the center of the axis having radius *r* and thickness *d*_r_.

## Results and discussions

### Characterization of synthesized CNTs

**Fig. 3:**
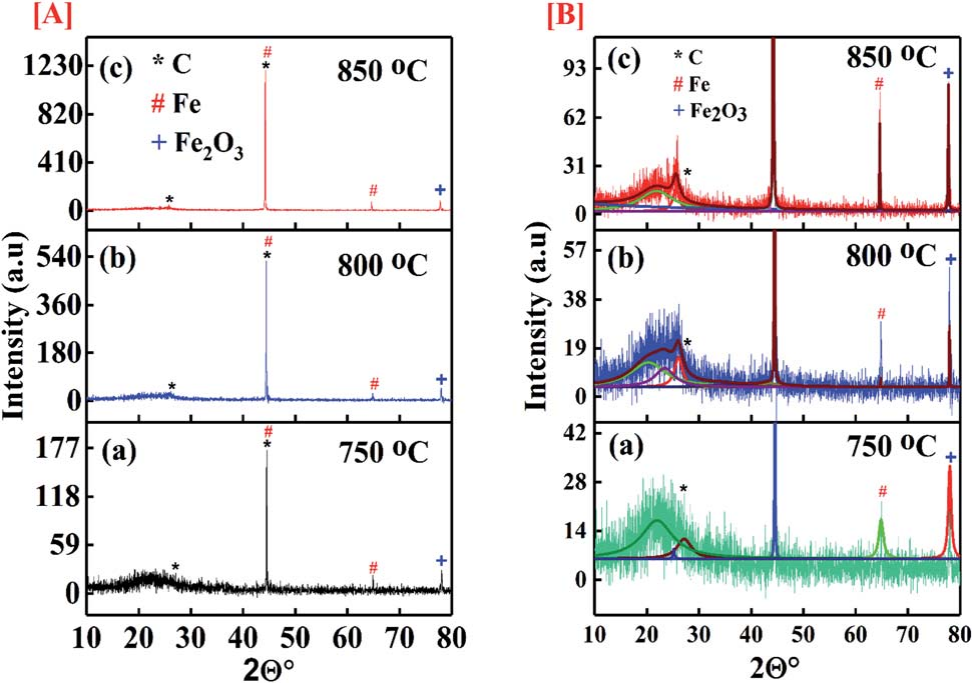
[A] Full XRD spectra of the CNMs synthesized at (a) 750 °C; (b) 800 °C; (c) 850 °C; [B] enlarged XRD spectra the CNMs synthesized at (a) 750 °C; (b) 800 °C; (c) 850 °C.

Fig. 3 represents the XRD spectra of the CNT samples grown at 750 °C, 800 °C, 850 °C on catalyst and show enhanced crystallinity. The broad peak at 2*θ*° between 20° to 30° is observed for the sample represents amorphous silica gel which was the content of the catalyst.^41–43^ The characteristic peak of graphitic carbon is observed at 2*θ*° of 27.14° and 43.5° corresponding to (002) and (100) planes of carbon [JCPDS card no. 75-1621]. The wider peak at 2*θ*° of 25.8° is persuaded by the disordered carbon inducing the defect levels in the atomic carbon structure. The presence of strong characteristic peak at 43.5° XRD spectra of all synthesized samples confirms successful deposition of carbon. The characteristics peak for crystalline metal Fe for the planes (110) and (002) are observed at 2*θ*° of 44.58° and 64.82° respectively [JCPDS card no. 87-0721] which could also be overlapping with that of the carbon peak at 43.5°. The spectra also show presence of unreduced Fe_2_O_3_ (JCPDS 33-0664) at 2*θ*° of 77.96°. Most of the iron oxide of the catalysts get reduced to pure Fe due to reduction by hydrogen gas. However, the amount of hydrogen used for reduction process was kept constant for all the samples. The reduction process at different temperatures was not expected to be same in all samples having leftover with some unreduced iron oxide as well.

**Fig. 4:**
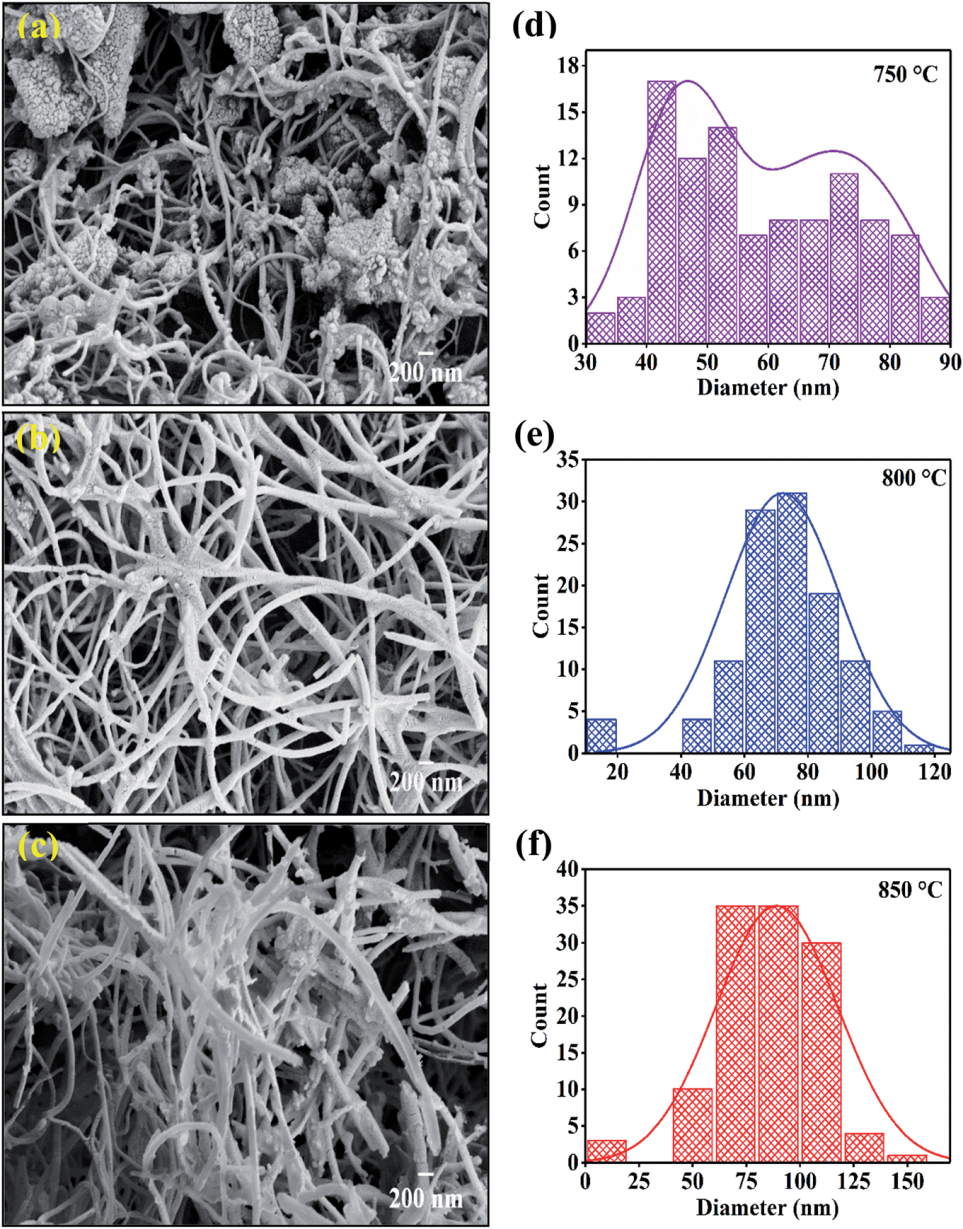
FESEM images of CNTs synthesized at various temperature on catalyst (a) 750 °C; (b) 800 °C; (c) 850 °C and (d), (e), (f) are respective diameter distribution of CNTs.

The field enhanced scanning electron microscopic (FESEM) images of synthesized carbon nanomaterials at 750 °C, 800 °C and 850 °C are shown in Fig. 4(a–c). Here, in all the synthesized samples, tubular nanostructures were observed. The CNTs were found agglomerated on the surface of the catalysts. The yield of nanotubes sample synthesized at temperature 750 °C showed very less yield while synthesized sample at temperature 800 °C and 850 °C showed high yield of nanotube formation. CNTs of different morphologies were observed. The forest like structure with no orientation in any direction found in FESEM images. The helical shape is also observed due to the pairing of pentagonal and heptagonal (P–H) carbon rings which arranged themselves in a periodical manner within the hexagonal carbon network.^44^

Fig. 4(d–f) shows the diameter distribution of CNTs which are synthesized at 750 °C, 800 °C and, 850 °C. The diameter of CNTs is calculated from FESEM images with the help of ImageJ software. The sample deposited at 750 °C showed bimodal distribution having the diameter peaked at 45 nm and 75 nm which get converted to single mode distribution of the CNM diameter with increase in temperature. Results indicate that the average diameter of CNTs is increased with respect to increasing temperature. The highest average diameter (80 nm) is found in sample synthesized at 850 °C. The increase in outer diameter of the CNMs depends on the thermodynamics involved in the pyrolytic decomposition of xylene solution at different temperatures.

**Fig. 5:**
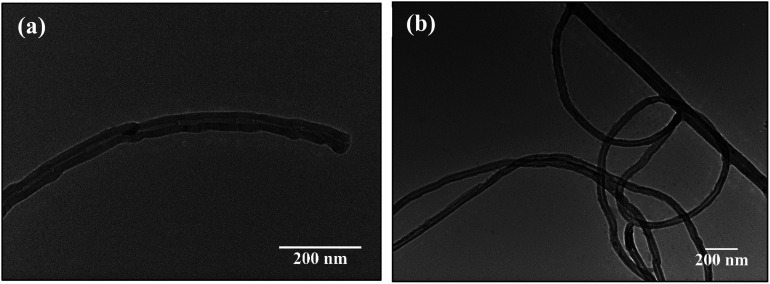
HRTEM images of CNTs synthesized at temperature of (a) 750 °C, (b) 800 °C.

More detailed analysis of the different structures of CNTs synthesized at different temperatures was done through HRTEM and presented in Fig. 5(a and b). Fig. 5(a) confirms the formation of hollow MWCNT having bamboo like structure. The metal catalyst is still present inside the tube showing Fe diffusion. Fig. 5(b) shows hollow structure undergoing secondary growth. The secondary growth was supposed to be initiated from unreacted metal catalyst particles that were left on the surface of the CNTs grown *via* tip-growth mechanism. The catalyst responsible for the secondary growth was probably trapped on surface of the growing CNTs which gives a growth site for secondary CNTs. FESEM images confirmed the increase in outer diameter of the CNTs with increase in pyrolytic temperature. This is attributed to the increase in number of layers of the CNTs. The increase in carbon layers cause increase in number of edge sp^3^-type carbon atoms, giving more curvature and increase in structure disorder. The defects incorporated in the CNTs formed were further evidenced by Raman spectroscopy in the following section.

**Fig. 6:**
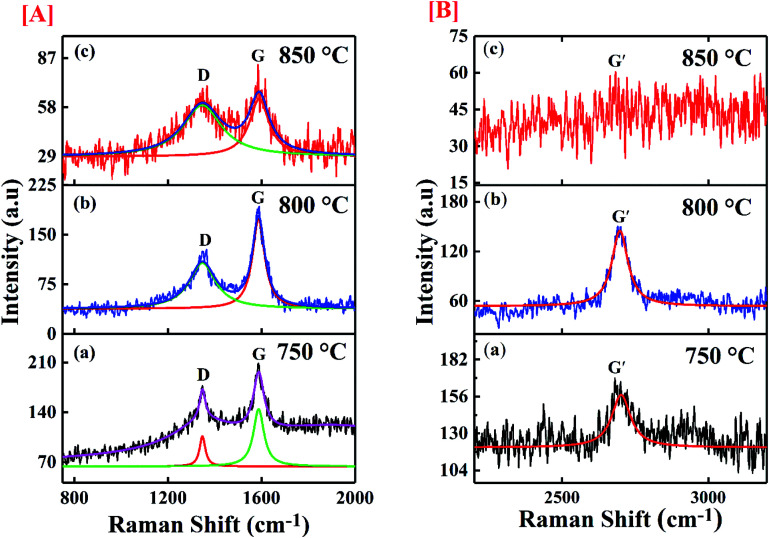
Image of the Raman spectra of synthesized materials at [A] (a) 750 °C; (b) 800 °C; (c) 850 °C with scale range 750 cm^−1^ to 2000 cm^−1^, [B] (a) 750 °C; (b) 800 °C; (c) 850 °C with scale range 2200 cm^−1^ to 3200 cm^−1^.

The molecular conformation for the synthesized CNTs was done through Raman spectroscopy and presented in Fig. 6. The figure shows the de-convoluted Raman spectra for the samples. The spectrum was taken in the range of 100 cm^−1^ to 3200 cm^−1^ by using an argon laser which has 514 nm excitation wavelength. The two main characteristic graphitic bands are seen in the spectrum of CNTs at 1580 cm^−1^ and 1350 cm^−1^. The band at 1580 cm^−1^ (G band) is assigned to graphitization structure due to ordered carbon arrangement in a materials and it is initiated from the first order Raman scattering of CNTs. It is initiated from vibration of the in-plane C–C bond and non-dispersive in nature. The D band at 1350 cm^−1^ represents defective graphite like materials which occurs due to the existence of disorder in carbon atom arrangement in the materials and it originates from second order Raman scattering.^45–47^ This band is dispersive and appears at 1350 cm^−1^ for 2.41 eV (514.5 nm) laser energy which shifts by 50 cm^−1^ eV^−1^ with change in probing energy of laser used. The D and G bands show shift in their positions in comparison to their original values. Table 2 shows the shifted values of the bands. A shift greater than ±2 cm^−1^ signifies the defect induced in the sample. The D band shows a shift of 15 cm^−1^ and 17 cm^−1^ for the samples synthesized at 800 °C and 850 °C respectively. The shift is associated with the strain induced in C–C bonds of the carbon atoms whereas, the G band shows up shift of 6, 10 and 5 cm^−1^ for samples deposited at 750 °C, 800 °C and 850 °C respectively. The upshift of the G band is related to the chemical, structural and doping induced defects in the samples.

**Table 2:** The *I*_D_/*I*_G_ ratio of synthesized materials on catalyst at different temperatures

Pyrolysis temperature (°C)	% weight of carbon deposition	*I*_D_	*I*_G_	*I*_D_/*I*_G_
750	22.16	1348.00	1586.57	0.84
800	59.92	1365.20	1590.16	0.85
850	92.00	1367.32	1585.64	0.86

In addition, the spectrum shows a band positioned around 2700 cm^−1^ named as G′ band which corresponds to the number of the layers of graphene contributing to CNT formation. The ratio (*I*_D_/*I*_G_) of the intensity of D (defect) and G (graphitization) bands is a noble indicator of the quality of carbon samples. Table 2 gives the details of the intensity ratio and other parameters. The maximum defect has been observed for the sample synthesized at 850 °C, whereas the sample deposited at 800 °C shows minimum defects. The defect level in the CNTs plays important role in the binding efficiency of the structure. The experimental results of MWCNTs like chirality, diameter *etc.* were further used to estimate the binding affinity of the CNTs towards SARS-CoV-2 virus.

### Interaction study of SWCNT and MWCNTS with SARS-COV-2

#### Molecular docking studies

**Fig. 7:**
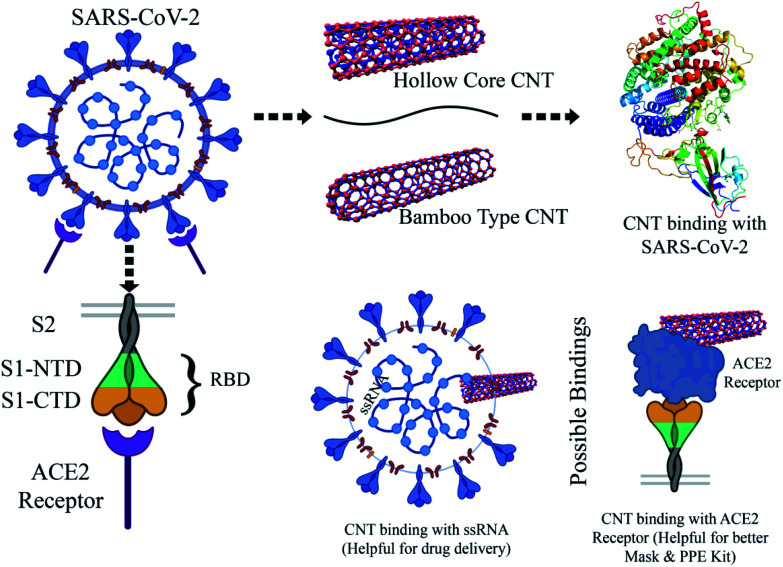
Schematic for understanding the interaction between MWCNT and SARS-CoV-2.

**Fig. 8:**
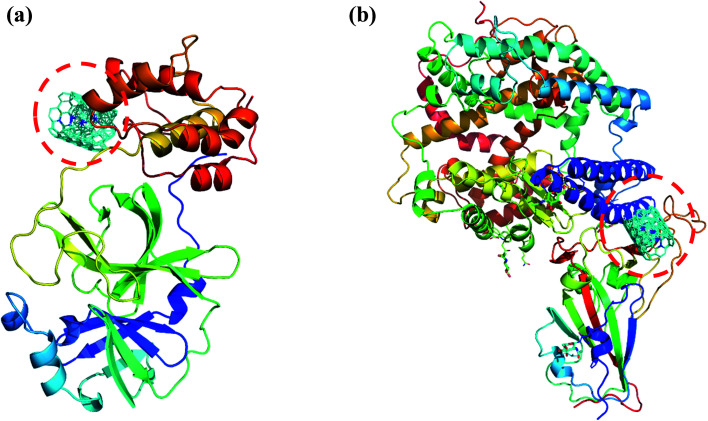
(a) Docked structure of MWCNT with 6LU7 (b) a docked structure with 6LZG. Color coding of 3D structure are as follows: ASP, GLU – bright red; CYS, MET – yellow; LYS, ARG – blue; SER, THR – orange; PHE, TYR – mid blue; ASN, GLN – cyan; GLY – light grey; LEU, VAL, ILE – green; ALA – dark grey; TRP – purple; HIS – pale blue; PRO – flesh; others – tan.

**Fig. 9:**
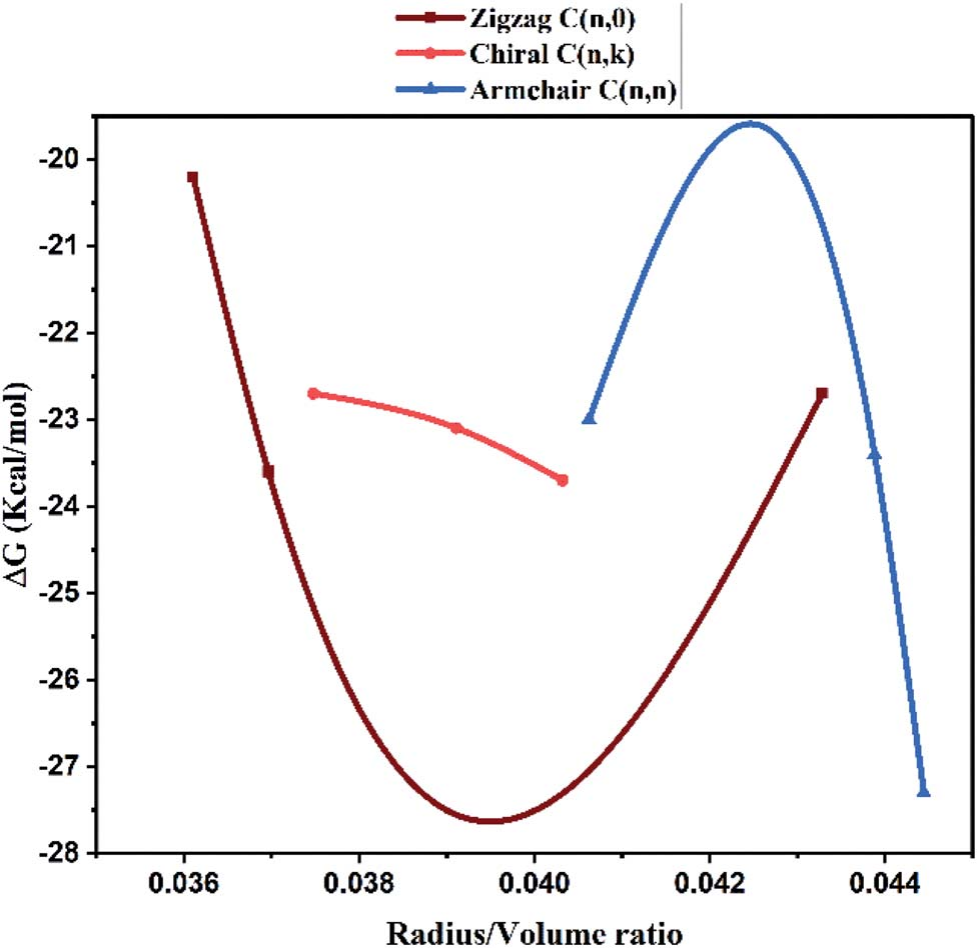
Correlation between the radius to volume ratio of HMWCNT and the binding energy within the active site of SARS-CoV-2.

The structure function correlations of the synthesized MWCNTs in terms of binding SARS-CoV-2 virus were established. The binding affinity of the synthesized MWCNTs with SARS-CoV-2 was investigated through molecular docking technique based on experimental data. SARS-CoV-2 is a corona virus with spike protein consisting of RBM (receptor-binding motif), NTD, (N-terminal domain); FP (fusion peptide); the core structure (β1, β2, β3, β4, β5, β6, β7) disulfide bonds (C480–C488; C336–C361; C391–C525) and ACE2 domains for receptor binding. Each domain of SARS-CoV-2 has specific residues in the active sites for binding. The 3D structure analysis in molecular docking results have shown that various MWCNT structure are accommodated to one of the six binding site pockets. In the present work, the binding modes of MWCNTs with SARS-CoV-2 were examined. The performed *in silico* docking study was based on two parameters: (1) different chirality of MWCNTs like armchair, zigzag, chiral and (2) variable radius to volume ratios. The parameters used in the molecular docking were extracted from Fig. 4 and 5 of FESEM and HRTEM images respectively. The docking studies reveal presence of different binding pocket site within SARS-CoV-2 for interaction of MWCNT. Fig. 7 shows the schematic details of interaction mechanism between MWCNTs and SARS-CoV-2. The highest binding energy was shown by the armchair MWCNT (both hollow-core MWCNT and bamboo type MWCNT) in comparison with the chiral and zigzag structure. MWCNT in comparison with SWCNT gives the better docking result, which suggests that multiple wall of the nanotube as well as chirality plays an important role in nanotube binding reactivity with the SARS-CoV-2.^48,49^ The complete docking result is shown in Table 3, which represents the binding sites and the type of interactions between SARS-CoV-2 and MWCNTs (Fig. 8). It is interesting to note that with increasing radius to volume ratio of MWCNTs, the binding energies of MWCNT also increases for all three (zigzag, armchair and chiral) types of MWCNTs. The increase in binding energy could be correlated with the structure and morphology induced surface reactivity of the CNTs. FESEM and HRTEM data reveals that the increase in outer diameter is due to the increase in number of coaxial carbon layers/walls inside the tubes. The increase in carbon layers is associated with increase in number of edge sp^3^-type carbon giving more curvature and increased defect levels thereby increasing the surface reactivity. Moreover, the Raman spectra (Fig. 6) show that the defect level increases for MWCNTs with increasing diameter. The significant up shift of the D (>15 cm^−1^) and G (>5 cm^−1^) band with increasing diameter also contributes to enhanced surface reactivity for the CNTs as explained in the preceding section. We can infer from these results that by increasing radius to volume ratio of MWCNT, the probability of its interaction with amino acids within the active site of SARS-CoV-2 will increase and the binding affinities (Fig. 9). We also analyzed the type of interaction between MWCNT and SARS-COV-2, which could be helpful for the understanding of drug binding mechanism. The list of ACE2 and RBD residues are given in Table 4. ACE2 can form hydrophobic and strong electrostatic (including π–π, and π–cation) interactions with the binding domain of ATR1. For ACE2 receptor, the SARS-CoV-2 tyrosine backbones, even though present on a loop, are mutually stabilized by hydrogen bonding and the side chains are locked in place by a π–π aromatic interactions between the phenyl rings. This enables both these tyrosine side-chains to form a strong electrostatic contact with Thr27 side chain of ACE2. The role of glycine in both SARS-CoV-2 and RBD is to provide a motif for capturing ACE2, while other residues of RBD like Y449/Y453/Y473 are used for strong electrostatic and π–cation interaction. The viral spike RBDs mimics these interactions to gain entry *via* strong non-covalent attachment. For this interaction, the SARS-CoV-2 spike RBD uses a combination of a π–cation interaction and a strong electrostatic interaction to bind with Y449/Y453/Y473 while the disulfide bond and the binding receptors of core structure show the π–cation and π–π interaction. Hence, the synthesized MWCNTs has the strongest interaction with SARS-CoV-2, and it can alter the structure and function of SARS-CoV-2 with higher accuracy.

**Table 3:** Molecular docking results for the interactions of SWCNT and MWCNTs with SARS-CoV-2

Type of MWCNT	List of CNT structure	Highest binding energy (Δ*G*) (kcal mol^−1^)		Binding site			
				6LU7		6LZG	
		6LU7	6LZG	Binding residues	Type of interaction	Binding residues	Type of interaction
Hollow-core CNT	C (4,0)	−22.7	−23.4	RBM	Hydrophobic, π–cation	ACE2	Hydrophobic, π–cation, π–π
	C (4,2)	−22.7	−24.4	RBM	Hydrophobic, π–cation	ACE2	Hydrophobic, π–cation, π–π
	C (4,4)	−23.0/−12.2^a^	−27.1	RBM	Hydrophobic, π–cation	Fusion peptide	Hydrophobic, π–cation
	C (5,0)	−20.2	−25.2	CORE (β2, β4), RBM	Hydrophobic, π–π, π–cation	ACE2	Hydrophobic, π–cation, π–π
	C (5,3)	−23.1	−28.3	RBM, (C480–C488)	Hydrophobic, π–cation	RBM, FP	Hydrophobic, π–cation
	C (5,5)	−23.4/−12.6^a^	−29.5	RBM, (C480–C488)	Hydrophobic, π–cation	ACE2	Hydrophobic, π–cation, π–π
	C (6,0)	−23.6	−25.7	RBM	Hydrophobic, π–cation	ACE2	Hydrophobic, π–cation, π–π
	C (6,3)	−23.7	−26.3	RBM, (C480–C488)	Hydrophobic, π–cation, π–π	RBM	Hydrophobic, π–cation
	C (6,6)	−27.3/−12.6^a^	−27.2	RBM, di sulphide bond (C480–C488)	Hydrophobic, π–cation, π–π	Di sulphide bond (C480–C488)	Hydrophobic, π–π
Bamboo type CNT	C (4,0)	−20.2	−22.7	RBM	Hydrophobic, π–cation	ACE2	Hydrophobic, π–cation, π–π
	C (4,2)	−21.3	−23.3	RBM	Hydrophobic, π–cation	ACE2	Hydrophobic, π–cation, π–π
	C (4,4)	−22.9	−25.4	RBM	Hydrophobic, π–cation	Fusion peptide	Hydrophobic, π–cation
	C (5,0)	−20.3	−22.8	RBM	Hydrophobic, π–cation	ACE2	Hydrophobic, π–cation, π–π
	C (5,3)	−21.8	−24.2	RBM	Hydrophobic, π–cation	RBM, FP	Hydrophobic, π–cation
	C (5,5)	−22.7	−27.4	RBM, (C480–C488)	Hydrophobic, π–cation	ACE2	Hydrophobic, π–cation, π–π
	C (6,0)	−23.5	−23.9	RBM	Hydrophobic, π–cation	ACE2	Hydrophobic, π–cation, π–π
	C (6,3)	−23.9	−24.3	(C480–C488)	Hydrophobic	RBM, (C480–C488)	Hydrophobic, π–cation
	C (6,6)	−26.5	−29.2	RBM, di sulphide bond (C480–C488)	Hydrophobic, π–cation, π–π	Di sulphide bond (C480–C488)	Hydrophobic, π–cation, π–π

a Molecular docking study performed with single walled carbon nanotube.

**Table 4:** RBD and ACE receptor's residue names

Strain	RBD residue	ACE2 residue
SARS-CoV-2	K417	D30
	Y449	D38, Q42
	Y453	H34
	Y473	T27
	F486	M82/L79/Q24
	Y489	K31
	Q493	K31/H34/E35
	T500	Y41
	N501	Y41/K353/G354
	G502	Q325/K353/G354

Furthermore, we have also investigated the binding affinities of SWCNT of same morphology and structure with SARS-CoV-2 virus for the comparison. The results are presented in Table 3. Interestingly, the MWCNTs showed far greater (more than double) binding affinity of −29 kcal mol^−1^ with the 6LZG and −27.3 kcal mol^−1^ for 6LU7 motif as compared to SWCNTs showing only −12.6 kcal mol^−1^ for both the motifs. In addition to enhanced binding energy of the MWCNTs, the morphology of the synthesized nanotubes could also be explained to prevent the attached virus from cell internalization.^50^ The motifs 6LZG and 6LU7 which showed maximum binding with the MWCNTs, are predicted to be responsible for cell internalization. Binding with MWCNTs would passivate these active sites for further interaction with the cells for uptake. Moreover, the HRTEM images show the length of the MWCNTs to be in micron size. The morphology of the MWCNTs, being one dimensional and of much longer size would prohibit internalization due to phagocytosis.^27^ Based on the molecular docking results, the highest binding energy was shown by the armchair MWCNT (both hollow-core MWCNT and bamboo type MWCNT) in comparison with the chiral and zigzag structure. Therefore, only armchair HMWCNT and BMWCNT was selected for the MD simulation and free binding energy calculations with SARS-CoV-2 main protease and ACE2 receptor.

#### Molecular dynamic simulation

**Fig. 10:**
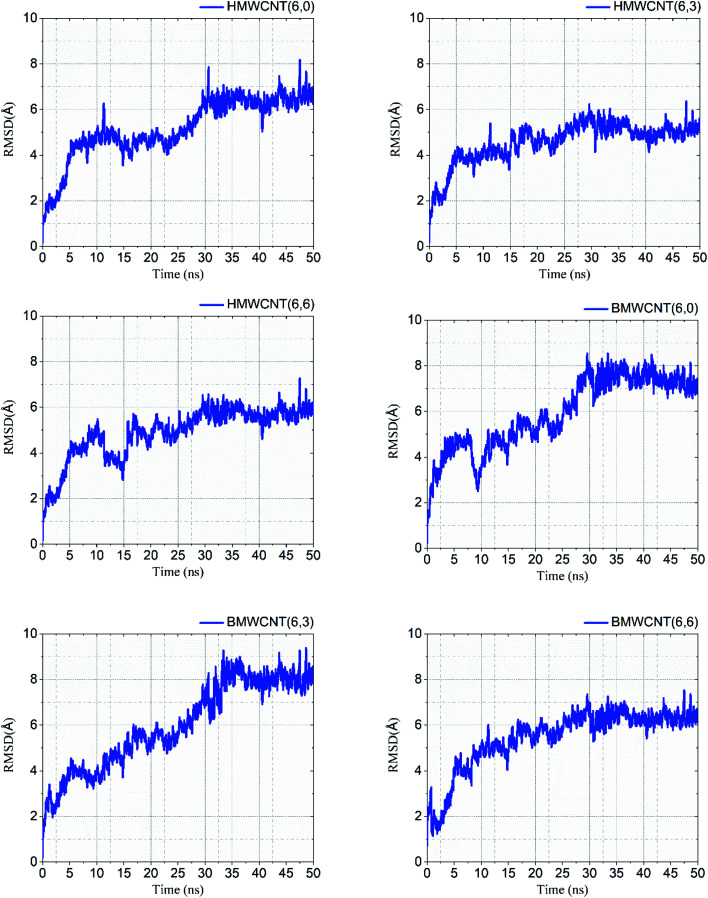
RMSD values of all simulated HMWCNT and BMWCNT complex structure with SARS-CoV-2 spike RBD ACE2 receptor.

We performed atomistic simulations of hollow and bamboo shaped MWCNT with different chirality of armchair (6,6), chiral (6,3) and zigzag (6,0) structure. The MD simulation provides insight into the effect of structure and chirality of MWCNTs with SARS-CoV-2 spike RBD complexed ACE2 receptor. Interestingly, the structure and chirality of MWCNTs affect the dynamic behavior of structure, thermodynamic properties, and radial distribution probability of SARS-CoV-2 spike RBD complexed ACE2 receptor. We analyzed the RMSD values in order to investigate the structural and conformational stability of all six simulated complex structure. RMSD values of all six simulated complex structure was shown in Fig. 10 and the structural and conformational stabilities for the dynamics of all six complex systems were calculated in terms of RMSD and plotted in Table 5.

**Table 5:** RMSD values of the simulated complex structure

CNT structure	RMSD (Å)	
	Hollow-core MWCNT	Bamboo type MWCNT
C (6,0)	6.78	7.42
C (6,3)	5.55	8.53
C (6,6)	5.50	6.36

Our results from the MD simulation inferred that the SARS-CoV-2 spike RBD ACE2-complex with the armchair chirality of both HMWCNT and BMWCNT have least RMSD values of 5.50 Å and 6.36 Å respectively depicting higher stability in terms of electrostatic interactions and overall conformational stability of the simulated complex structure. The root mean square deviation (RMSD) conveys the information of overall stability of the SARS-CoV-2 ligand complex in terms of deviation from the initial structure. As shown in Table 5 all the six systems were significantly stable with variable deviation. Armchair and zigzag structure however, remained significantly stable throughout the simulation with negligible fluctuation as compared to chiral systems inferring the formation of most stable ligand bounded protein complex of simulated system followed by a stable RMSD value. The data indicates that all the systems showed stable internal motion. In Fig. 10, the RMSDs values of the complex systems of SARS-CoV-2 with HMWCNT were equilibrated in the last 20 ns for the armchair and zigzag structure. For the chiral structure, the simulated complex structure was equilibrated in the last 15 ns. The 50 ns long simulation shows more reliability and preciseness towards the stability and equilibration of the conformational changes of the simulated complex structure. The bamboo shaped MWCNT, comparatively indicated larger fluctuations of the RMSD values than HMWCNT.

**Fig. 11:**
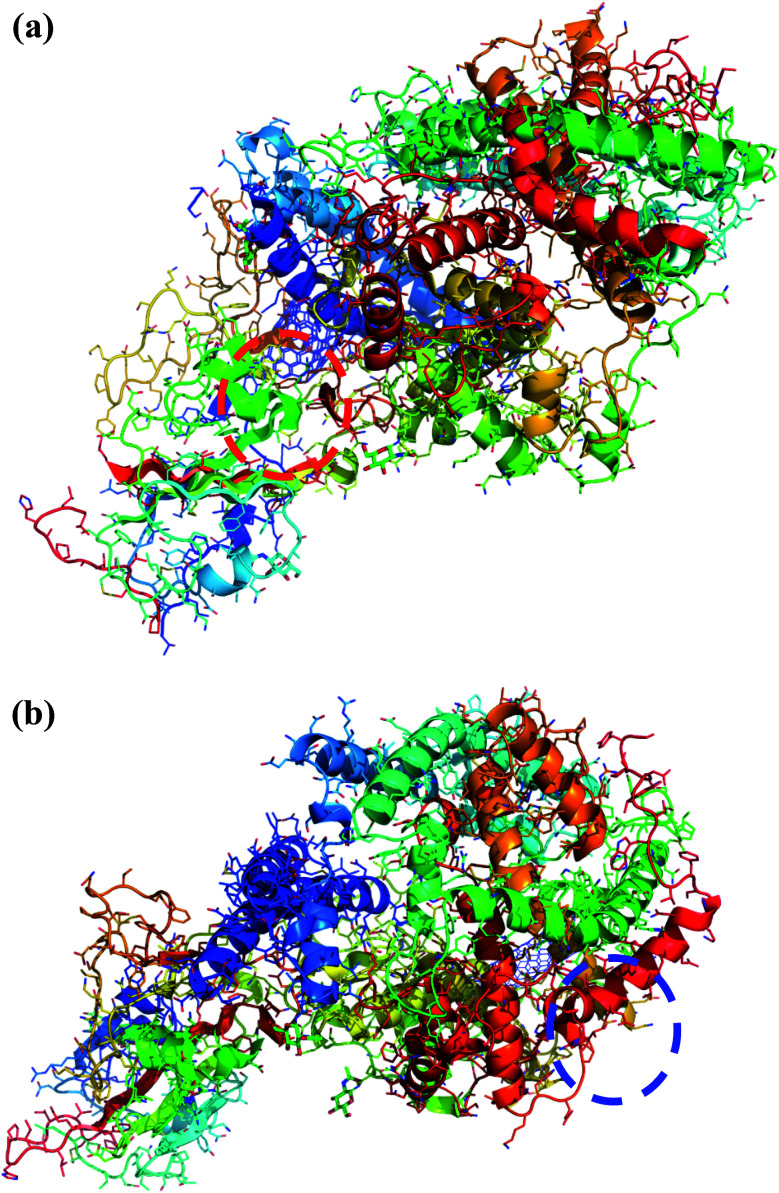
(a) 3D structure of simulated HMWCNT complex structure with SARS-CoV-2 spike RBD ACE2 receptor. Color coding of 3D structure are as follows: ASP, GLU – bright red; CYS, MET – yellow; LYS, ARG – blue; SER, THR – orange; PHE, TYR – mid blue; ASN, GLN – cyan; GLY – light grey; LEU, VAL, ILE – green; ALA – dark grey; TRP – purple; HIS – pale blue; PRO – flesh; others – tan. (b) 3D structure of simulated BMWCNT complex structure with SARS-CoV-2 spike RBD ACE2 receptor. Color coding of 3D structure are as follows: ASP, GLU – bright red; CYS, MET – yellow; LYS, ARG – blue; SER, THR – orange; PHE, TYR – mid blue; ASN, GLN – cyan; GLY – light grey; LEU, VAL, ILE – green; ALA – dark grey; TRP – purple; HIS – pale blue; PRO – flesh; others – tan.

**Fig. 12:**
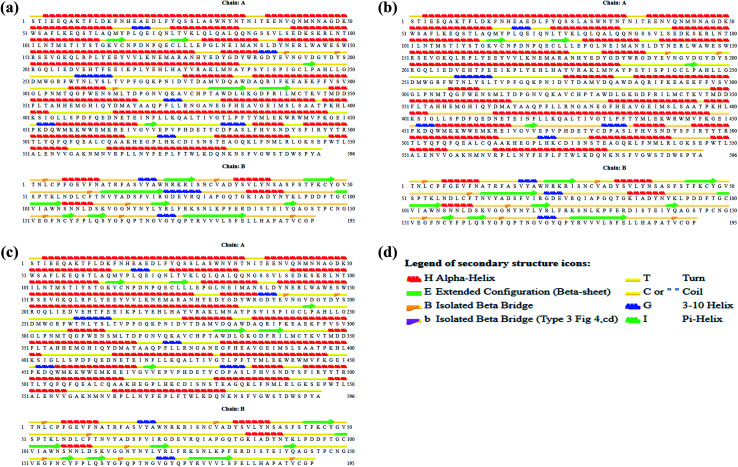
(a) Secondary structure of SARS-CoV-2 spike RBD ACE2 receptor. (b) Secondary structure of simulated complex structure of SARS-CoV-2 spike RBD ACE2 receptor with HMWCNT (6,6). (c) Secondary structure of simulated complex structure of SARS-CoV-2 spike RBD ACE2 receptor with BMWCNT (6,6). (d) Secondary structure color notation.

**Fig. 13:**
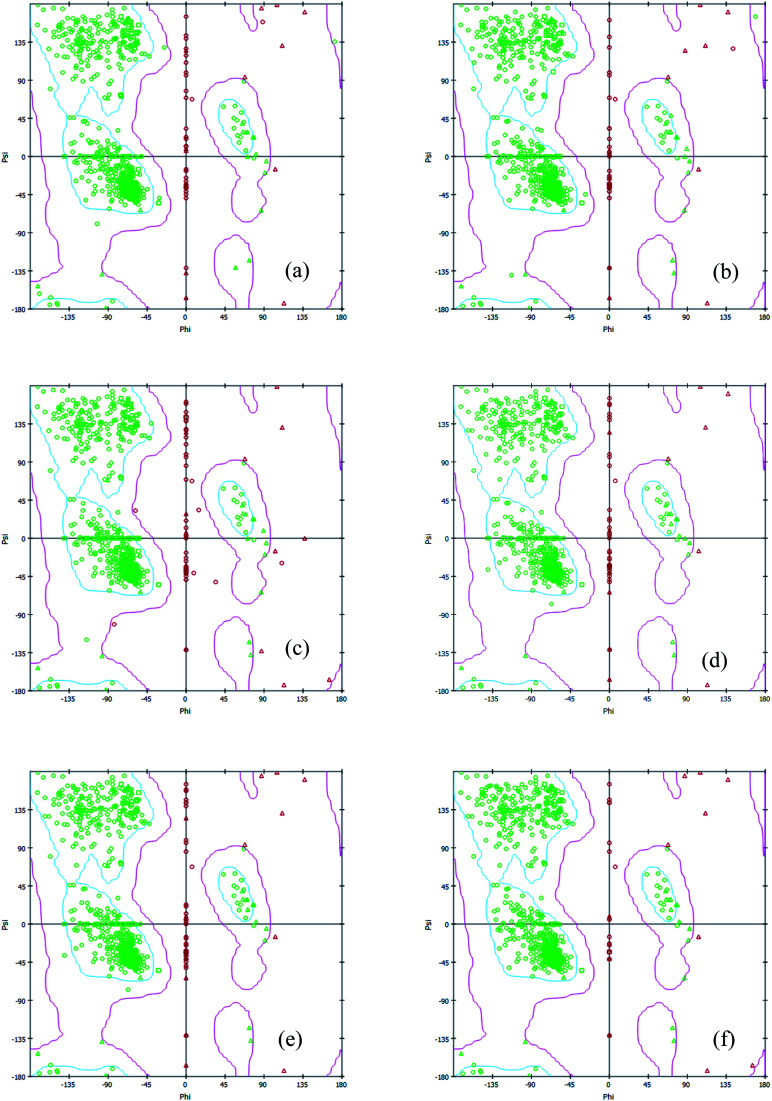
2D Ramachandran plot of all simulated complex structure of SARS-CoV-2 spike RBD ACE2 receptor with (a) HMWCNT (6,0) (b) HMWCNT (6,3) (c) HMWCNT (6,6) (d) BMWCNT (6,0) (e) BMWCNT (6,3) (f) BMWCNT (6,6).

Interestingly, the armchair (6,6) based complex simulated structure showed more stable dynamic behavior than zigzag and chiral structure of both hollow and bamboo shaped MWCNT. Remarkably, the higher stability of armchair structure can be explained by the trajectory analysis which confirms that by increasing the radius to volume ratio of MWCNT, the probability of its interaction and stability of conformational changes also get affected. The large fluctuations in RMSD values represents the large conformational or structural changes with repositioning of the atomic coordinates inside the binding site resulting in different structural changes (3D structure of simulated condition Fig. 11(a) and (b)). By analyzing trajectory of each simulated model, the changes in the atomic coordinates of SARS-CoV-2 structure also reflects the conformational changes in the secondary structure of the SARS-CoV-2 spike RBD complexed ACE2 receptor. To gain the insight knowledge of the effect of simulation on the secondary structure the STRIDE (secondary structure identification interface) program was used to see the conformational changes in the amino acid residues (Fig. 12(a–c)).^51^ The secondary structure of simulated complex structure shows significant conformational and structural changes during the MD simulation process. Further, Ramachandran plot was plotted in 2D to analyze the changes in the coordinates of the simulated system. We analyzed and confirmed the changes in the conformational structure of all SARS-CoV-2–ligand complexes for the energetically activated coordinates region for the backbone dihedral angles *ψ* against *φ* of the amino acid residues. Validation of simulated system using Ramachandran plot (Fig. 13) were performed with the PROCHECK server.^52^ The validation data is tabulated (Table 6) in terms of most favored region followed by additional allowed regions, generously allowed region and the disallowed regions.

**Table 6:** Validation data of Ramachandran plot for simulated complex system through PROCHECK server

	Hollow-core MWCNT			Bamboo type MWCNT		
	C (6,0)	C (6,3)	C (6,6)	C (6,0)	C (6,3)	C (6,6)
Residues in most favoured regions [A,B,L]	88.9%	89.8%	90.0%	90.6%	90.6%	91.1%
Residues in additional allowed regions [a,b,l,p]	9.6%	9.1%	8.3%	9.1%	9.1%	8.6%
Residues in generously allowed regions [∼a,∼b,∼l,∼p]	0.8%	0.6%	1.1%	0.0%	0.0%	0.0%
Residues in disallowed regions	0.8%	0.5%	0.6%	0.3%	0.3%	0.3%
*G* factor	−0.10	−0.04	−0.01	−0.10	−0.10	−0.12

**Fig. 14:**
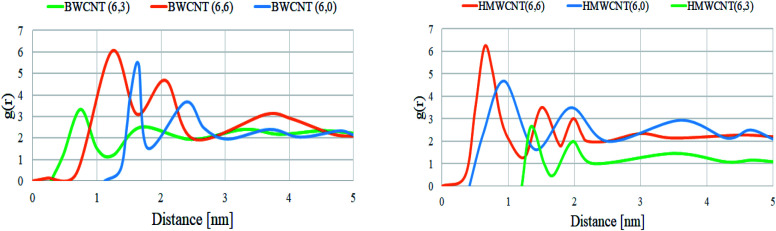
Radial distribution function of all MWCNT with respect to the SARS-CoV-2 simulated complex structure.

By the trajectory analysis, we also analyzed and computed the radial distribution function (RDF) with respect to the principal axis of the H/B MWCNT for the carbon atoms of all simulations (Fig. 14). In statistical mechanics, the radial distribution function, in a system of particles, describes the density variation as a function of distance from a reference particle for the study of inter-particle interactions of the MWCNT and SARS-CoV-2.

A pronounced ring-like multilayer structure for HMWCNT is clearly visible, with a strong first peak of the RDF followed by up to about four further maxima for the armchair chirality. The amplitudes of all further maxima and minima decrease exponentially with the distance from the MWCNT. This shows the density variation of MWCNT as a function of distance from a reference particle of SARS-CoV-2. In accordance with recent simulations comparing different chirality, structure and surface to volume ratio with SARS-CoV-2, this is a consequence, first, of the type, MWCNT interaction with SARS-CoV-2 spike RBD ACE2 receptor. Analyzing the radial distribution curves in contrast to the SARS-CoV-2 spike RBD ACE2 receptor, the RDFs of the armchair of both hollow and bamboo shaped MWCNT structure showed a strong dependence on the chirality of the MWCNT. For armchair and zigzag MWCNTs, the RDFs show a common trend (Fig. 14). The density at the edge of the MWCNT increases with the length of the MWCNT. The hollow MWCNT interacts strongly with SARS-CoV-2 spike RBD ACE2 receptor in terms of the electrostatic and van der Waals interaction. While, BMWCNT interacts weakly under electrostatic and van der Waals interaction with SARS-CoV-2 spike RBD ACE2. This effect is represented in the RDF as a peak in the region where, *r* is smaller than the radius of the MWCNT. We also analyzed the thermodynamic parameters of all simulated complex systems. For assessing the binding affinity for particular ligand and receptor only molecular docking study are insufficient as these docking studies does not shows the conformational or structural changes occurring during the binding process with ligands. The all atomistic MD simulation covers the complete conformational and structural changes occurring during receptor–ligand interactions. By analyzing the receptor–ligand van der Waals and electrostatic interactions, MD simulation results were quite essential to justify the calculated binding affinity through molecular docking studies. The performed MD simulation shows the thermodynamics of the binding energy of ACE2 receptor based corona virus with different chiral structures of hollow and bamboo shaped MWCNT. We performed molecular mechanics generalized Born/surface area (MM/GBSA) analysis to estimate the binding energy of the all simulated structure. The free binding energies were calculated as a summation of basic four thermodynamic parameters Δ*E*_electrostatic_, Δ*E*_vdw_, Δ*G*_solv_ and solvent-accessible surface area (SASA). In our calculations, we were more concerned with the relative changes of the binding affinity with respect to chirality and surface to volume ratio of both HMWCNT and BMWCNT. Five thousand snapshots without water and organic molecules as well as chloride ions were extracted from the MD trajectories in the last 30 ns at 20 ps intervals for free binding energy analysis. Table 7 lists the calculated free binding energy in Δ*G* for each chirality of both HMWCNT and BMWCNT. The higher negative Δ*G* values as shown in Table 7 represent the higher adsorption rate of the SARS-CoV-2 spike RBD ACE2 receptor on the respective MWCNT. It also shows that the binding energy of BMWCNT is significantly lesser than that in the HMWCNT which indicates much weaker adsorption of SARS-CoV-2 spike RBD ACE2 receptor on the BMWCNT. The result is also consistent as per the molecular docking results in terms of chirality. Where the order of binding affinity was Δ*G*_armchair_ > Δ*G*_zigzag_ > Δ*G*_chiral_. The stronger binding affinity of armchair MWCNT of both hollow and bamboo shaped is reconfirmed by the thermodynamic study of the simulated complex structure.

**Table 7:** The binding free energies for SARS-CoV-2 with HMWCNT and BMWNT of different chirality complex systems by MM-GBSA method

Energies (kcal mol^−1^)	SARS-CoV-2 (6LZG)					
	HMWCNT (6,0)	HMWCNT (6,3)	HMWCNT (6,6)	BMWCNT (6,0)	BMWCNT (6,3)	BMWCNT (6,6)
Δ*E*_electrostatic_	−68.41 ± 0.30	−62.03 ± 0.02	−65.18 ± 0.10	−53.03 ± 0.01	−43.8 ± 0.88	−51.3 ± 0.12
Δ*E*_vdw_	−89.18 ± 0.16	−84.32 ± 0.73	−92.55 ± 0.43	−71.34 ± 0.12	−67.01 ± 0.56	−76.41 ± 0.18
Δ*G*_GB_	49.81 ± 0.11	45.61 ± 0.82	51.14 ± 0.06	39.21 ± 0.25	35.0 ± 0.84	34.25 ± 0.71
Δ*G*_SA_	−11.03 ± 0.65	−17.6 ± 0.14	−13.47 ± 0.27	−10.02 ± 0.73	−14.03 ± 0.20	−11.24 ± 0.01
Δ*H*	−8.32 ± 0.28	−11.02 ± 0.32	−6.37 ± 0.09	−9.21 ± 0.82	−19.32 ± 0.14	−16.24 ± 0.26
−*T*Δ*S*	37.14 ± 0.31	41.21 ± 0.54	32.97 ± 0.16	35.09 ± 0.62	36.22 ± 0.76	38.24 ± 0.30
Δ*G*	−81.67 ± 0.81	−77.13 ± 0.57	−87.09 ± 0.02	−60.09 ± 0.55	−53.62 ± 0.38	−66.46 ± 0.58

## Conclusions

In summary, the interaction between SARS-CoV-2 and synthesized MWCNTs of different morphology has been investigated. The effect of MWCNT chirality on the binding efficiency of SARS-CoV-2 virus was studied with respect to two different essential components of the SARS-CoV-2 structure *i.e.*6LU7 (COVID-19 main protease) and 6LZG (spike receptor-binding domain complexed with its receptor ACE2). Our MD simulation studies showed the effectiveness of MWCNT in terms of RDF and thermodynamic properties for the adsorption of SARS-CoV-2 spike RBD ACE2 receptor. Here, we can also infer that the armchair structure for both hollow and bamboo shaped MWCNT structure show a strong binding or adsorption affinity for the SARS-CoV-2 spike RBD based ACE2 receptor. The RDF data also gives understanding about the direct correlation of the density of MWCNT with the length of the MWCNT for the adsorption of any biological entity. Further, an investigation *via* computational studies clear that glycine in both SARS-CoV-2 and RBD is to provide a motif for capturing ACE2, while other residues of RBD like Y449/Y453/Y473 are used for strong electrostatic and π–cation interaction. MWCNTs with core hollow morphology showed highest binding energy of −87.09 kcal mol^−1^ with the 6LZG which can be exploited in PPE kits to capture any free SARS-CoV-2 particles present in aerosol form, if any.

## Conflicts of interest

There are no conflicts to declare.

## Supplementary Material
